# Candidate Obesity Biomarkers Identified Through Multi‐Omics Analysis, Mendelian Randomization, and Mediation Analysis

**DOI:** 10.1002/fsn3.71803

**Published:** 2026-04-20

**Authors:** Mahan Si, Yongsheng Zhao, Yingting Wu, Wenli Zhao, Xuan Liu, Mengxi Jiang

**Affiliations:** ^1^ Department of Pharmacology, School of Basic Medical Sciences Capital Medical University Beijing China; ^2^ Department of Hepato‐Pancreato‐Biliary Surgery, First Medical Center Chinese PLA General Hospital Beijing China; ^3^ School of Pharmacy Capital Medical University Beijing China; ^4^ School of Traditional Chinese Medicine Capital Medical University Beijing China

**Keywords:** Mendelian randomization, obesity, omics

## Abstract

Obesity has become a major public health problem worldwide and a key driver of metabolic disorders. Metabolomics and lipidomics have enabled the discovery of circulating biomarkers, yet their causal relevance remains unclear. In this study, we profiled fasting serum samples from non‐obese and obese participants using untargeted ultra‐performance liquid chromatography–tandem mass spectrometry (UPLC‐MS/MS) metabolomics and liquid chromatography–tandem mass spectrometry (LC–MS/MS) lipidomics to identify obesity‐associated metabolites and lipids, and prioritized candidates by integrating two‐sample bidirectional Mendelian randomization (MR) and MR‐based mediation analyses with immune cell traits based on publicly available genome‐wide association studies (GWAS) summary statistics. Receiver operating characteristic (ROC) analyses and regression‐based association analyses were further performed to evaluate discriminatory performance and relationships with clinical parameters. We identified hexanoylglycine and lysophosphatidylcholine (LPC (16:0)) as significantly altered in obesity. Both biomarkers showed consistent MR evidence suggesting potential causal relevance to obesity and exhibited associations with obesity status and body mass index (BMI). Two‐step MR further suggested that immune cell traits might partially mediate these relationships. In addition, these biomarkers were associated with blood pressure measures, a key indicator of cardiometabolic risk. Collectively, our findings highlight hexanoylglycine and LPC (16:0) as candidate serum biomarkers linked to obesity.

## Graphical abstract

Untargeted metabolomics and lipidomics integrated with two‐sample bidirectional Mendelian randomization identified hexanoylglycine and LPC (16:0) as obesity‐associated biomarkers. Hexanoylglycine and LPC (16:0) showed potential causal links to BMI, were partially mediated by immune cell traits, and were associated with blood pressure, indicating relevance to cardiometabolic risk.

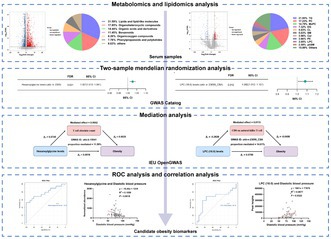

## 1 Introduction

Obesity has become a global health crisis, driving a wide spectrum of metabolic disorders, including type 2 diabetes, cardiovascular disease, and certain types of cancer, thereby reducing both quality of life and life expectancy (Blüher 2019). Identifying reliable biomarkers is essential for advancing prevention, diagnosis, and treatment strategies.

Metabolomics and lipidomics enable systematic biomarker discovery and have revealed distinct metabolic and lipidomic profiles in obesity (Ammar et al. 2017; Guleken et al. 2023; Men et al. 2016). For example, branched‐chain and aromatic amino acids, glutamine, and fatty acids have been reported to be altered in obese individuals (Boden 2008; Jennings et al. 2016; Park et al. 2024). Recent large‐scale lipidomics studies further suggest characteristic obesity‐related lipid signatures, such as altered ceramides and lysophospholipids (Chew et al. 2019; Huang et al. 2024; Rauschert et al. 2015). However, findings across studies remain inconsistent, and most evidence is observational, which limits causal interpretation and mechanistic understanding.

To move beyond association, MR uses genetic variants as instruments to test genetically proxied relationships between exposures and outcomes (Davies et al. 2018). Recent metabolome‐wide MR analyses have also been used to identify metabolites linked to adiposity‐related traits, providing complementary evidence to observational profiling (Hughes et al. 2024). In addition, obesity is increasingly recognized as a chronic low‐grade inflammatory state in which immune cells play central roles (Schleh et al. 2023; Shaikh et al. 2023; Soták et al. 2024). Whether immune‐mediated pathways contribute to metabolic and lipid alterations in obesity is unclear.

Prior metabolomics, lipidomics, and MR studies in obesity have largely been conducted separately, and immune trait mediation has rarely been explored. Here, we integrate untargeted metabolomics and lipidomics measured in clinical samples with two‐sample MR and MR‐based mediation analyses using immune cell traits as potential mediators, thereby linking multi‐omics biomarkers to obesity risk and suggesting immune‐related pathways for follow‐up. We report hexanoylglycine and LPC (16:0) as serum biomarkers associated with obesity, and our MR‐based mediation analyses suggest that immune cell traits may partially mediate these associations.

## 2 Materials and Methods

### 2.1 Study Design

**FIGURE 1:**
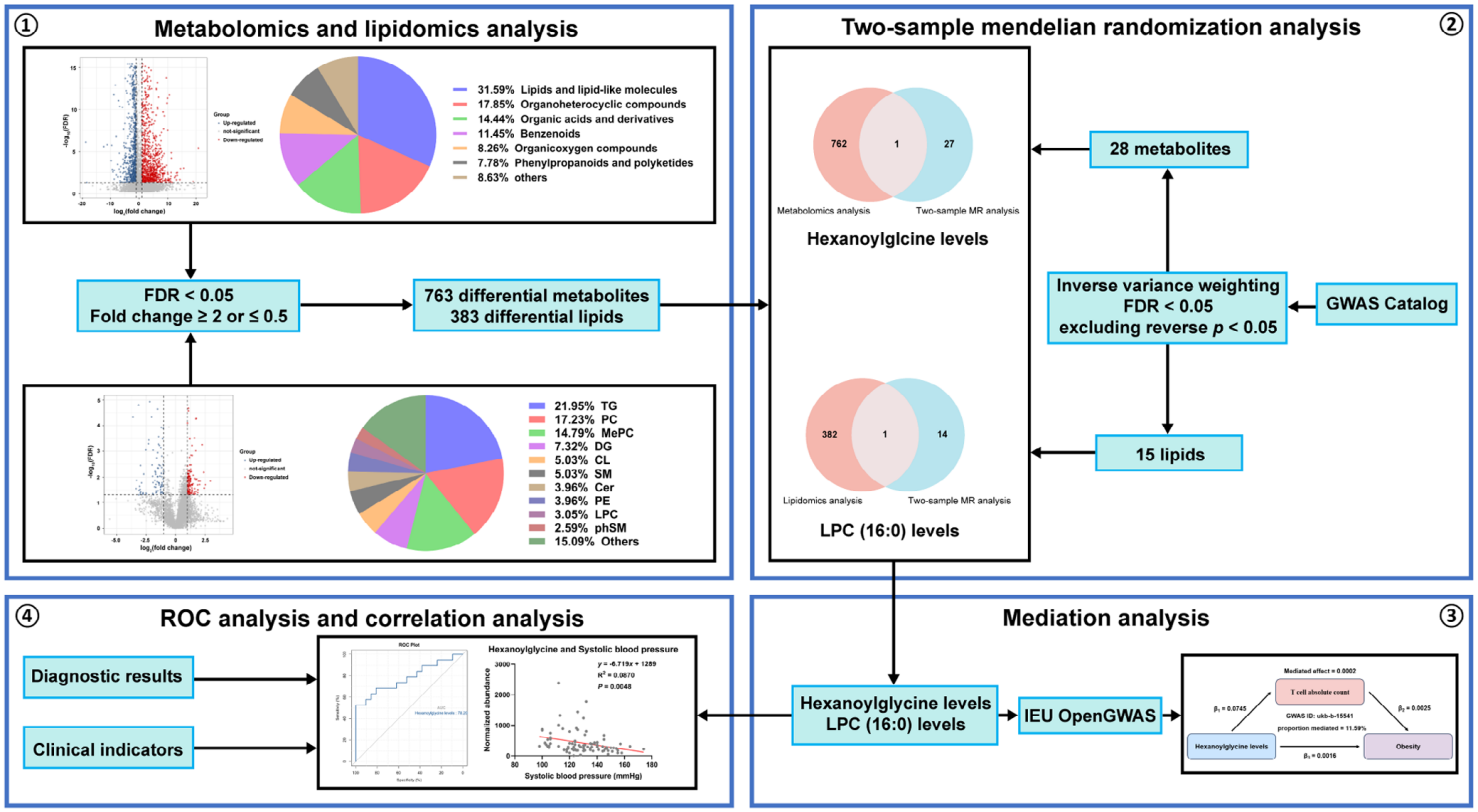
Flowchart of the study.

We performed untargeted metabolomic and lipidomic analyses using serum samples from non‐obese or obese individuals. The detailed group design and sample information are provided in the participants and sample collection and analysis sections. Publicly available GWAS data on serum metabolites, lipids, immune cell types, and obesity were then integrated. Two‐sample MR was used to evaluate potential causal relationships between metabolites, lipids, and obesity, thereby validating our experimental findings. To further explore mediation, two‐step MR analyses were performed with immune cells as intermediates. All MR procedures followed the STROBE‐MR guidelines (Skrivankova et al. 2021). ROC analysis was used to assess diagnostic potential, and correlation analyses were used to examine associations between biomarker levels and metabolic disease parameters (Figure 1).

### 2.2 Participants

A total of 117 participants (81 males, 36 females; aged 22–86 years) were recruited from the Chinese PLA General Hospital. Participants were classified according to BMI as non‐obese (BMI < 30.0 kg/m^2^; including normal‐weight and overweight individuals) or obese (BMI ≥ 30.0 kg/m^2^), using the WHO definition (Wharton et al. 2020). Individuals with underweight BMI (< 18.5 kg/m^2^) were excluded. We did not further subdivide the non‐obese group due to limited sample size for stable estimates in subgroup analyses. Exclusion criteria included acute infection, decompensated chronic disease, pregnancy or lactation. Age distribution is summarized in Table 1, and cohort characteristics by BMI are displayed in Table 2. Blood pressure was measured three times in the morning using a standard sphygmomanometer by trained staff, and the average value was recorded. The study was approved by the Ethics Committee of the Chinese PLA General Hospital (S‐2024‐134‐01). Written informed consent was obtained from all participants or their legal guardians. Group differences in gender were tested using chi‐square analysis, while other clinical variables were compared by *t*‐test.

**TABLE 1:** The age distribution of the participants.

Age range (years)	Non‐obese	Obese	*p*
22–30	5	11	0.6920
31–40	6	7	0.3627
41–50	7	7	0.9327
51–60	16	18	0.4637
61–70	14	12	0.7855
71–80	5	5	0.2127
81–86	1	3	NA

**TABLE 2:** Characteristics of the study cohort.

	Non‐obese	Obese	*p*
Sex (male/total)	37/54	44/63	0.8772^a^
Age (years)	54.59 ± 14.87	52.08 ± 16.81	0.3971
Weight (kg)	64.86 ± 8.72	92.47 ± 12.20	< 0.0001
Height (cm)	169.41 ± 7.41	168.73 ± 8.28	0.6444
BMI (kg/m^2^)	22.55 ± 2.18	32.41 ± 2.87	< 0.0001
Systolic blood pressure (mmHg)	120.89 ± 10.91	139.98 ± 16.17	< 0.0001
Diastolic blood pressure (mmHg)	72.22 ± 8.72	85.66 ± 10.72	< 0.0001

*Note:* Data were shown as mean ± SD.

^a^ The *p‐*value was obtained from the chi‐square test.

### 2.3 Sample Collection and Analysis

Participants fasted overnight before morning venous blood collection. Serum was obtained by centrifugation at 3000 rpm for 10 min at 4°C, aliquoted into pre‐chilled tubes, and stored at −80°C for biochemical, metabolomic, and lipidomic analyses. Fasting glucose was measured using a Cobas 8000 analyzer (Roche Diagnostics, Rotkreuz, Switzerland) in the Clinical Laboratory of the Chinese PLA General Hospital.

### 2.4 Untargeted Metabolomics by UPLC‐MS/MS

For sample preparation, 20 μL serum was mixed with 60 μL methanol, vortexed for 15 min, and centrifuged at 13,000 rpm for 15 min at 4°C. Supernatants were diluted for analysis. Chromatographic separation was performed on an ACQUITY HPLC BEH C18 column (2.1 × 100 mm, 1.7 μm; Waters, Milford, MA, USA) with solvent A (0.1% formic acid in water) and solvent B (0.1% formic acid in acetonitrile) at 0.4 mL/min and 35°C. Mass spectrometry was conducted using the SYNAPT G2‐Si high‐definition mass spectrometer (Waters, Milford, MA, USA) with electrospray ionization, which provides a typical resolution of > 40,000 FWHM full (width at half maximum) at m/z 556. Leucine enkephalin (LE) was used as the lock mass for data acquisition (m/z 556.2771 in positive mode and m/z 554.2615 in negative mode). Data were processed using Progenesis QI (Waters) for peak alignment and baseline correction, followed by built‐in normalization prior to statistical analysis. Metabolites were putatively annotated by matching against the Human Metabolome Database (HMDB) via ChemSpider. Orthogonal partial least squares discriminant analysis (OPLS‐DA) was used for visualization of group separation. The OPLS‐DA models suggested separation between groups (R^2^Y (cum) = 0.95, Q^2^ (cum) = 0.92) and in the negative ion mode (R^2^Y (cum) = 0.95, Q^2^ (cum) = 0.72). Permutation testing was not performed; therefore, OPLS‐DA results were interpreted descriptively and differential features were defined using univariate testing with Benjamini‐Hochberg false discovery rate (FDR) and fold‐change thresholds. Differential metabolites were defined as FDR‐adjusted *q*‐value < 0.05 and fold change ≥ 2 or ≤ 0.5 (Wang et al. 2024). Quality control involved excluding features with excessive missing values (Sun and Xia 2024). After filtering, 54 non‐obese and 57 obese samples remained for analysis.

### 2.5 Lipidomics Analysis

Serum lipids were extracted using a modified liquid–liquid extraction with dichloromethane and methanol (Cequier‐Sánchez et al. 2008; Wu et al. 2024). Briefly, 30 μL serum was mixed with 100 μL methanol, vortexed (10 min, 4°C), followed by the addition of 190 μL dichloromethane and 60 μL water, vortexed again, and centrifuged (13,000 rpm, 10 min, 4°C). The lower (non‐polar) phase was collected, dried under nitrogen, and reconstituted in 100 μL acetonitrile:isopropanol:water (65:35:5). After centrifugation, supernatants were transferred to vials for analysis. LC–MS/MS was performed on a Q Exactive HF‐X mass spectrometer (Thermo Fisher Scientific, Waltham, MA, USA) coupled to an Ultimate 3000 LC system using an Acclaim RP LC 120 C18 column (2.2 μm, 2.1 × 150 mm; Thermo Fisher Scientific, Waltham, MA, USA). Injection volume was 10 μL; flow rate 0.4 mL/min; column temperature 55°C *.* The mobile phases were: (A) 40% acetonitrile/60% water with 0.1% formic acid and ammonium formate, and (B) 90% isopropanol/10% acetonitrile with the same additives. Data were acquired in both positive and negative ionization modes (resolution: 120,000 for full MS; 30,000 for MS/MS), with routine calibration to maintain mass accuracy within ±5 ppm. Data were processed using LipidSearch 4.2 (Thermo Fisher Scientific, Waltham, MA, USA) (Yamada et al. 2013). OPLS‐DA was used to visualize overall separation between groups and was interpreted descriptively only. To identify lipids with intergroup differences, Student's *t*‐tests were performed for each lipid feature. *P*‐values were adjusted for multiple testing using the Benjamini‐Hochberg FDR procedure. Lipid features were considered significant if FDR < 0.05, fold change ≥ 2 or ≤ 0.5 (Kim et al. 2024). Features detected in fewer than half of the samples in either group were removed, and samples with excessive missing values were excluded (Sun and Xia 2024), yielding 46 non‐obese and 50 obese participants for downstream analyses.

### 2.6 Metabolic Pathway Enrichment Analysis

Metabolic pathway analysis was performed using MetaboAnalyst 6.0 (Pang et al. 2024).

### 2.7 Data Sources of Two‐Sample MR Analysis

To explore potential causal relationships among circulating metabolites, lipids, obesity, and immune cells, we obtained GWAS summary statistics for obesity from large‐scale consortia. Detailed information on each dataset is provided in Table S1.

### 2.8 Data Sources of Mediation Analysis

Genetic variants associated with immune cells were obtained from the UK Biobank GWAS database (https://gwas.mrcieu.ac.uk/datasets).

### 2.9 Selection Criteria for Instrumental Variables (IVs)

IVs were restricted to genome‐wide significant single nucleotide polymorphisms (SNP) (*p* < 5e‐08). If unavailable, SNPs with *p* < 5e‐06 were considered. SNPs were pruned for linkage disequilibrium (*r* ^2^ < 0.001, 10,000 kb window) based on European samples from the 1000 Genomes Project (Abecasis et al. 2010). Missing exposure SNPs were replaced by linkage disequilibrium (LD) proxies, and palindromic or ambiguous SNPs were excluded (Hemani et al. 2018). The F statistic was calculated to assess instrument strength, with weak instruments (*F* < 10) excluded (Burgess and Thompson 2011). Instrument strength metrics (F‐statistics and variance explained) are summarized in Table S5 and Figure S2.

### 2.10 MR Analysis

MR analyses were conducted in R (v4.2.0) using the TwoSampleMR (v0.5.6), MRPRESSO, and mr. raps packages. Statistical power was estimated with the mRnd tool (https://cnsgenomics.shinyapps.io/mRnd), and PhenoScanner was queried to identify potential pleiotropic associations of instrumental SNPs. We performed two‐sample bidirectional MR to investigate genetically proxied relationships between serum metabolites, lipids, and obesity. Primary MR estimates were obtained using inverse variance weighting (IVW) and are reported with 95% confidence intervals (CIs). Results with FDR < 0.05 were considered statistically significant. To assess directionality, we conducted MR analyses in both directions using the same analytical framework; reverse‐direction results were reported and considered in interpretation. Sensitivity analyses included Cochran's Q test (heterogeneity), the MR‐Egger intercept (horizontal pleiotropy), MR‐PRESSO (outlier detection and correction), and leave‐one‐out analyses to assess robustness.

### 2.11 Mediation Analysis

Two‐step MR was applied to assess whether immune cells may mediate the putative effects of metabolites and lipids on obesity (Sanderson 2020). CIs for mediation estimates were obtained using the delta method (Carter et al. 2021). Robustness was evaluated using Steiger filtering (Hemani et al. 2017) for causal direction, Cochran's Q test and funnel plots (Tan et al. 2021) for heterogeneity, and MR‐Egger (Burgess and Thompson 2017) and MR‐PRESSO (Verbanck et al. 2018) for pleiotropy. Outliers were removed and causal estimates recalculated. If substantial heterogeneity persisted, random‐effects models were applied. Leave‐one‐out analyses further examined SNP influence. Results with *p* < 0.05 in heterogeneity or pleiotropy tests across any mediation step were excluded. Step‐specific MR estimates for each mediation path are provided in Table S6.

### 2.12 Venn Diagram

The intersections of differential serum metabolites or lipids between metabolomics or lipidomics analysis and two‐sample MR analysis were visualized as Venn diagrams using https://www.bioinformatics.com.cn(Tang et al. 2023).

### 2.13 Forest Plot

Associations between serum metabolites, lipids, and obesity derived from two‐sample MR were visualized as forest plots using https://www.bioinformatics.com.cn (Tang et al. 2023).

### 2.14 ROC Analysis

Samples with non‐missing values for both hexanoylglycine and LPC (16:0) were randomly split into training and testing sets at a 1:1 ratio using stratified sampling by obesity status. Classification was performed in R using the caret package, with a fixed random seed to ensure reproducibility. ROC curves were generated in the training set and evaluated in the testing set. Hiplot Pro (https://hiplot.com.cn/) was used for figure visualization.

### 2.15 Construction of the Composite Model

A composite diagnostic model based on hexanoylglycine and LPC (16:0) was built using binary logistic regression. Predicted probabilities were used for multivariable ROC analysis.

### 2.16 Correlation Analysis

Simple linear regression was used to evaluate the correlation between biomarker levels, systolic blood pressure (SBP), and diastolic blood pressure (DBP). The analyses were performed using GraphPad Prism 10.1.2.

### 2.17 Multivariable Regression Analysis

Multivariable regression models were used to evaluate associations of serum hexanoylglycine and LPC (16:0) with obesity status and blood pressure outcomes. Logistic regression was used for obesity status, adjusting for age and sex; biomarker concentrations were log_2_‐transformed and Z‐score standardized prior to modeling. Linear regression was used for SBP and DBP, adjusting for age, sex, and BMI; biomarker concentrations were analyzed on their original scale. Results are reported as odds ratios (ORs) per 1 SD increase for logistic models and as regression coefficients (β) per 1‐unit increase in biomarker concentration for linear models, with 95% confidence intervals and *p*‐values. Analyses were performed in R software.

### 2.18 Targeted LC–MS/MS Quantification of LPC (16:0)

Targeted quantification of LPC (16:0) was performed using an Agilent 1290 Infinity UPLC system (Agilent Technologies, Santa Clara, CA, USA) coupled to an AB Sciex TripleQuad 6500 Plus mass spectrometer equipped with a Turbo V electrospray ionization (ESI) source (Sciex, Framingham, MA, USA). Chromatographic separation was achieved on an Acclaim RSLC C18 column (2.2 μm, 2.1 × 150 mm; Thermo Scientific). The mobile phases consisted of (A) water/methanol/acetonitrile (3:1:1, v/v/v) and (B) isopropanol, both containing 5 mM ammonium formate. The flow rate was 0.3 mL/min with the following gradient: 0–1 min, 20% B; 1–4 min, 20%–80% B; 4–6 min, 80% B; 6–6.1 min, 80%–20% B; and 6.1–9 min, 20% B. The injection volume was 3 μL, and the column temperature was maintained at 45°C. Quantification was performed in positive‐ion multiple reaction monitoring (MRM) mode. The source parameters were set as follows: ion spray voltage, 5500 V; curtain gas, 35 psi; source temperature, 450°C; ion source gas 1, 50 psi; and ion source gas 2, 50 psi. The MRM transitions and collision energies for LPC (16:0) and the deuterated internal standard LPC‐d31 (16:0) are listed in Table S4.

## 3 Results and Discussion

### 3.1 Lipid‐Like Molecules Show the Most Pronounced Alterations in Obesity

**FIGURE 2:**
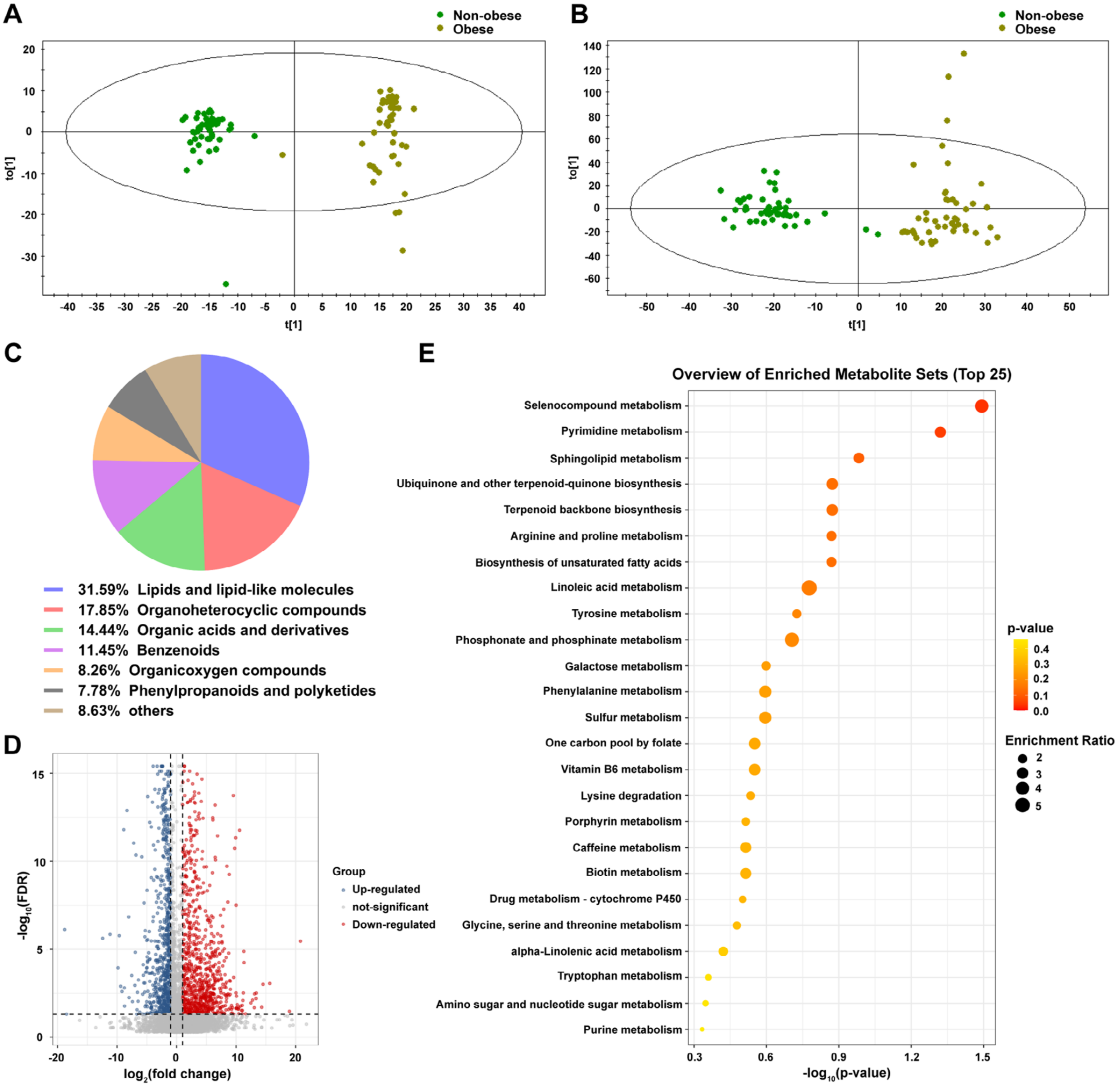
Untargeted metabolomic analysis of serum samples from non‐obese and obese participants. (A, B) OPLS‐DA plots show separation of serum samples between non‐obese and obese participants in positive and negative ion modes, respectively. (C) Classification and percentage distribution of differential compounds in metabolomics analysis results (*p* < 0.05). (D) Volcano plots show the differential metabolites between non‐obese and obese participants in metabolomics analysis (FDR < 0.05, fold change ≥ 2 or ≤ 0.5). (E) Overview of enriched sets of differential metabolites (FDR < 0.05, fold change ≥ 2 or ≤ 0.5) containing at least 5 entries using MetaboAnalyst 6.0 for KEGG enrichment.

To characterize metabolic differences between non‐obese and obese participants, we performed UPLC/MS‐based untargeted metabolomics. OPLS‐DA models revealed a clear separation between groups in both positive and negative ion modes (Figure 2A,B). In total, 1877 differential metabolites (*p* < 0.05) were identified, with lipid‐like molecules accounting for the largest proportion (31.59%), followed by organoheterocyclic compounds and organic acids (Figure 2C). Applying stricter criteria (FDR < 0.05, fold change ≥ 2 or ≤ 0.5), 763 significantly altered metabolites were retained (Figure 2D). Kyoto Encyclopedia of Genes and Genomes (KEGG) enrichment highlighted 25 major pathways (Figure 2E).

### 3.2 Differential Lipidomic Profiles in Obesity

**FIGURE 3:**
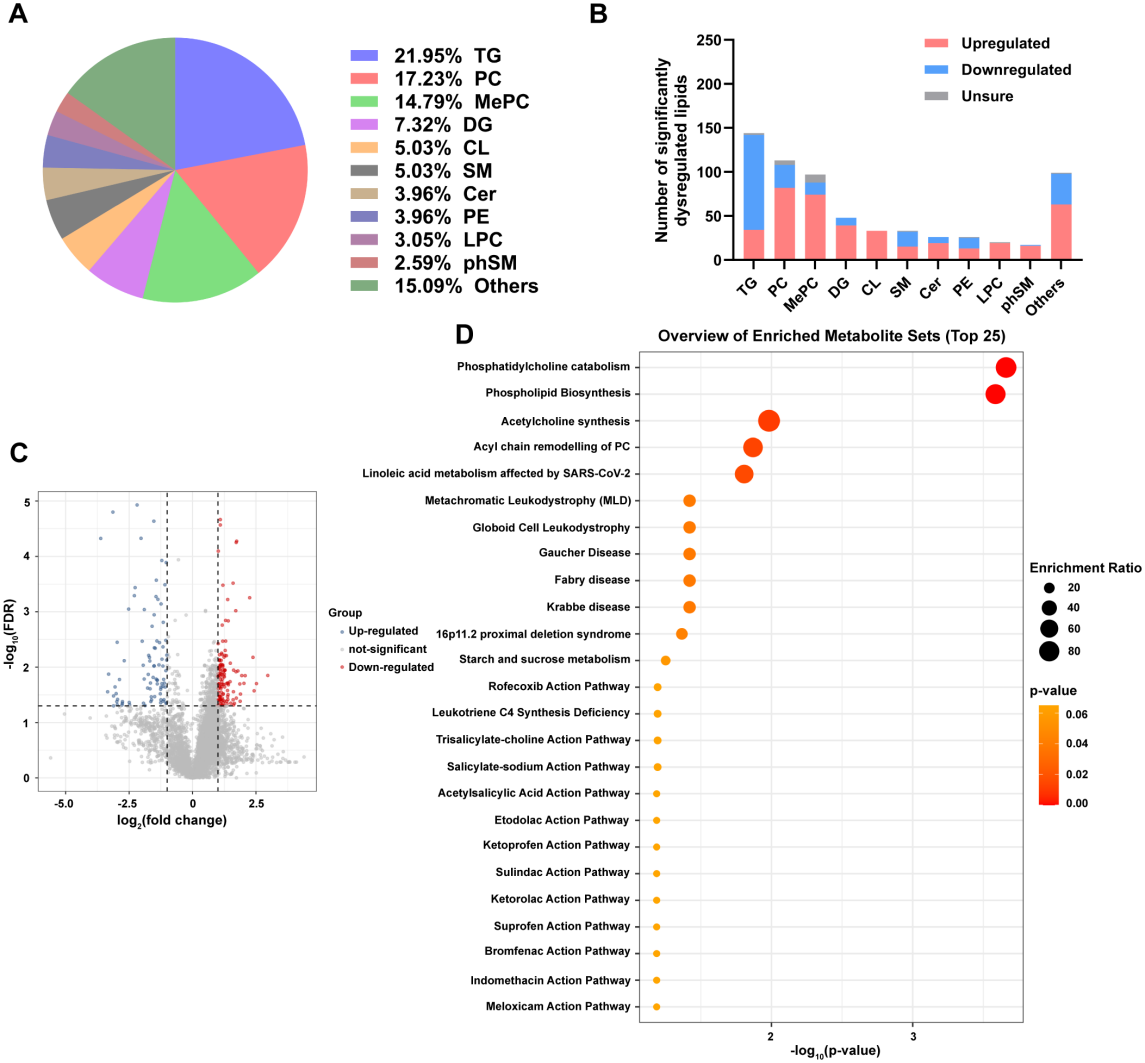
Lipidomics analysis of serum samples from non‐obese and obese participants. (A) Top 10 lipid categories with the highest proportion of altered lipids (*p* < 0.05). (B) Dysregulated lipids by lipid subclass (*p* < 0.05). (C) Volcano plots of the differential lipid ion peaks between non‐obese and obese participants calculated by peak height (FDR < 0.05 with fold change ≥ 2 or ≤ 0.5). (D) Overview of enriched sets of all the differential lipids (FDR < 0.05 with fold change ≥ 2 or ≤ 0.5) containing at least 5 entries using MetaboAnalyst 6.0 for RaMP‐DB enrichment.

We next performed lipidomics analysis on the same cohort. A total of 934 differential ion peaks were detected, corresponding to 656 unique lipids (*p* < 0.05). Triglycerides (21.95%), phosphatidylcholine (17.23%), and methyl phosphatidylcholine (14.79%) were the most altered subclasses (Figure 3A,B). After applying strict selection, 383 significantly altered lipid ions remained (Figure 3C). Relational database of Metabolomic Pathways (RaMP‐DB) enrichment revealed 25 enriched pathways (Figure 3D).

### 3.3 Genetic Evidence for an Association Between Hexanoylglycine and Obesity

**FIGURE 4:**
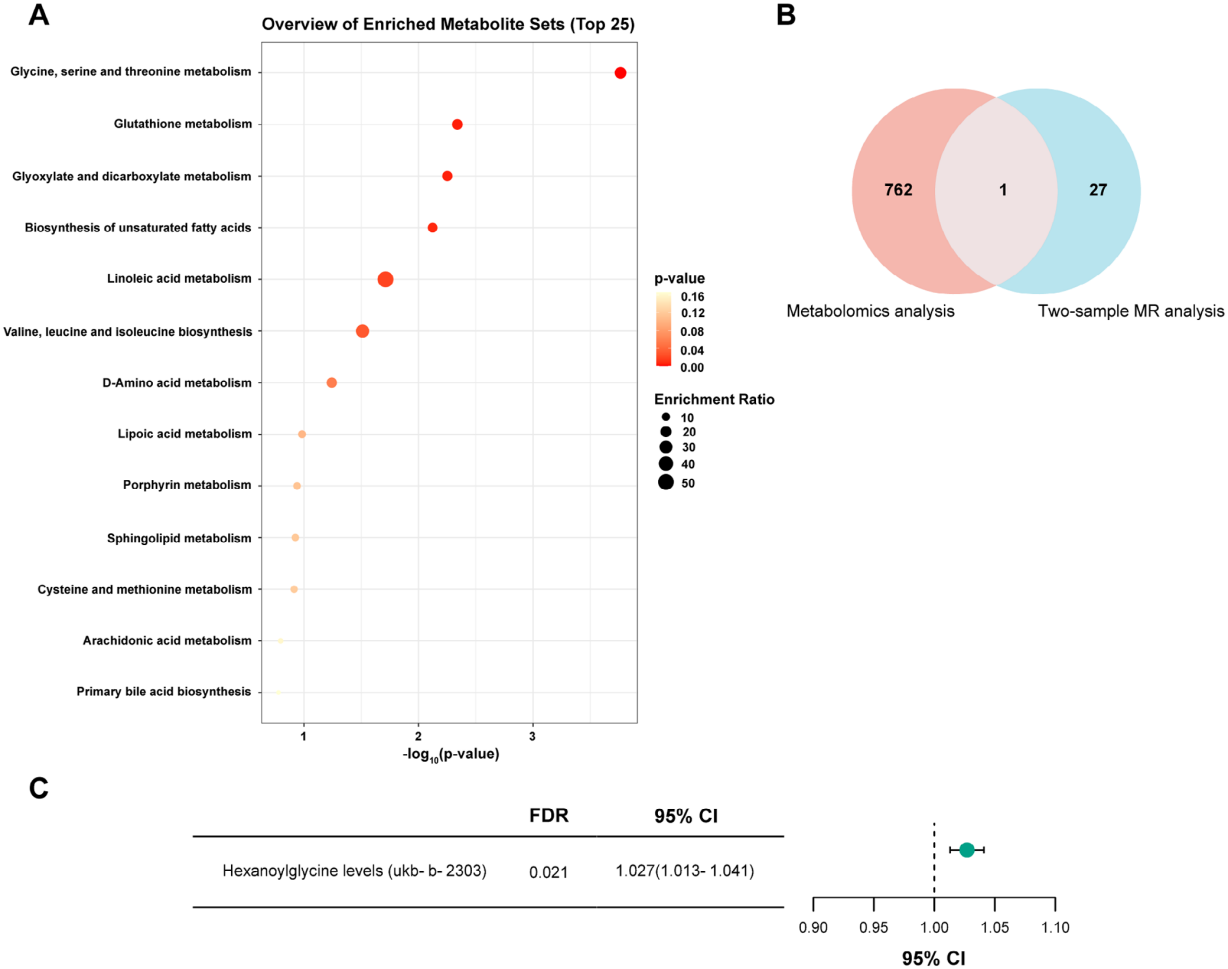
MR estimates of serum metabolites in relation to obesity. (A) Overview of enriched metabolite sets of serum metabolites in two‐sample MR analysis (FDR < 0.05) containing at least 5 entries using MetaboAnalyst 6.0 for KEGG enrichment. (B) Venn diagrams show the differential metabolites (FDR < 0.05, fold change ≥ 2 or ≤ 0.5) which are related to obesity in MR analysis (FDR < 0.05) as well. (C) Associations between genetically predicted hexanoylglycine levels and obesity traits in MR analyses. 95% CI represents the odds ratio.

To further explore this relationship, we conducted two‐sample MR analyses using obesity‐related GWAS traits. We identified 28 metabolite traits associated with obesity (FDR < 0.05) and assessed causal direction using bidirectional MR. Sensitivity analyses did not indicate substantial heterogeneity or horizontal pleiotropy. KEGG enrichment highlighted 13 pathways (Figure 4A). Among these, hexanoylglycine was downregulated in obesity and showed MR evidence suggesting potential causal relevance to obesity (Figure 4B,C). In multivariable logistic regression adjusted for age and sex, hexanoylglycine remained significantly associated with obesity (Table S2).

### 3.4 LPC (16:0) is an Obesity‐Associated Lipid Supported by MR and Targeted Quantification

**FIGURE 5:**
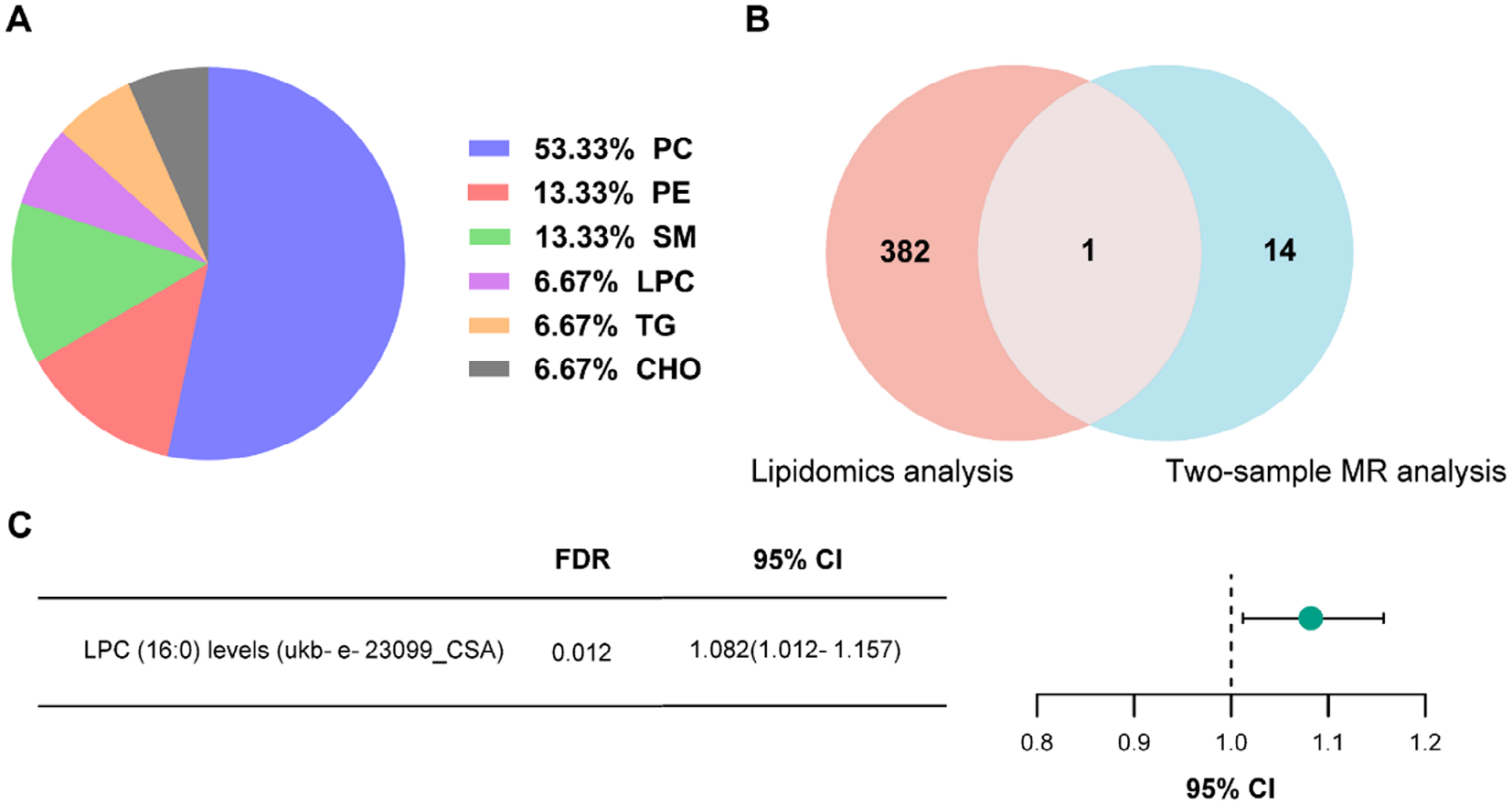
MR estimates of serum lipids in relation to obesity. (A) The categories by the proportion of relevant lipids to obesity studies in two‐sample MR analysis result (FDR < 0.05). (B) Venn diagrams of intersection between the differential lipids (FDR < 0.05 with fold change ≥ 2 or ≤ 0.5) in lipidomics analysis result and the relevant lipids in two‐sample MR study (FDR < 0.05). (C) Associations between genetically predicted LPC (16:0) levels and obesity traits in MR analyses. 95% CI represents the odds ratio.

In the MR analysis, 15 lipids were significantly associated with obesity (FDR < 0.05), mainly PCs (53.33%) (Figure 5A). LPC (16:0) overlapped between differential lipidomics and MR results and showed MR evidence suggesting potential causal relevance to obesity (Figure 5B,C). In multivariable logistic regression adjusted for age and sex, LPC (16:0) remained significantly associated with obesity (Table S2). To further validate the untargeted lipidomics result, we performed targeted LC–MS/MS quantification of LPC (16:0). The targeted analysis showed a consistent trend and supported the observed elevation of LPC (16:0) in the obese group (Figure S3).

### 3.5 Immune Cell Mediation of Hexanoylglycine's Effects

**FIGURE 6:**
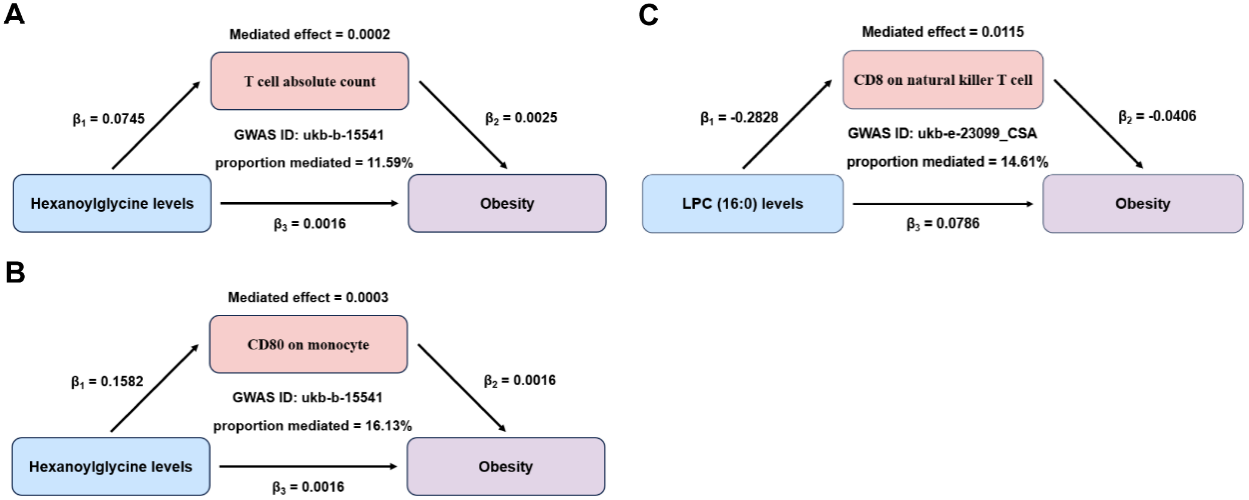
MR‐based mediation effects of serum hexanoylglycine and LPC (16:0) levels on obesity via immune cell traits. (A–C) The effects of serum hexanoylglycine and LPC (16:0) levels on obesity, which are mediated by immune cells, respectively.

We then investigated whether immune cells mediated the metabolite‐obesity relationship. Mediation analysis suggested that the effects of hexanoylglycine may be partly mediated by T cell absolute count (11.59%) and CD80 expression on monocytes (16.13%) (Figure 6A,B; Table 3). Detailed information on hexanoylglycine levels in metabolomics analysis results is shown in Table 4.

**TABLE 3:** The mediating effect of serum metabolites and lipids on obesity.

Metabolites	Mediator	Mediated proportion
Hexanoylglycine levels (ukb‐b‐15,541)	T cell absolute count	11.59%
Hexanoylglycine levels (ukb‐b‐15,541)	CD80 on monocyte	16.13%
LPC (16:0) levels (ukb‐e‐23099_CSA)	CD8 on natural killer T cell	14.61%

**TABLE 4:** The detailed information of hexanoylglycine and LPC (16:0) in metabolomics or lipidomics analysis results.

Metabolite or lipid ion	FDR	Fold change (obese/non‐obese)	Changes in obese participants
Hexanoylglycine	< 0.0001	0.4552	Downregulated
LPC (16:0)	0.0142	3.8353	Upregulated

### 3.6 Immune Cell Mediation of LPC (16:0)'s Effects

For LPC (16:0), mediation analysis revealed CD8 expression on natural killer T cells as the most significant mediator (14.61%) (Figure 6C and Table 3). Detailed information on LPC (16:0) in lipidomics analysis results is shown in Table 4.

### 3.7 Biomarker Potential of Hexanoylglycine and LPC (16:0)

**FIGURE 7:**
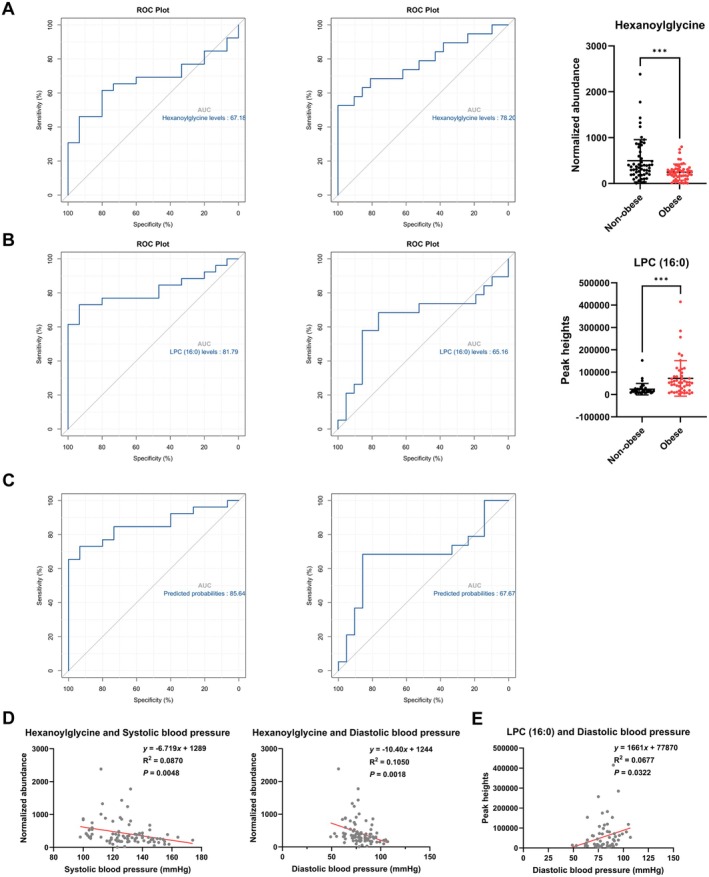
Identification of representative metabolites and lipids with good predictive ability for obesity and correlation with blood pressure. (A, B) ROC curves showing AUCs in the training set (left panel), testing set (middle panel), and biomarker levels in non‐obese versus obese participants (right panel). (C) ROC curves for the predicted probabilities obtained from the composite model with an AUC. (D, E) The correlation between serum hexanoylglycine and LPC (16:0) levels and systolic blood pressure and diastolic blood pressure, respectively. Statistical significance was assessed using Student's *t*‐test. ***p < 0.001.

To assess the discriminatory performance of hexanoylglycine and LPC (16:0) for obesity, ROC analyses were conducted using training and testing sets. Hexanoylglycine yielded areas under the curve (AUCs) of 0.67 in the training set and 0.78 in the testing set (Figure 7A). LPC (16:0) yielded AUCs of 0.82 in the training set and 0.65 in the testing set (Figure 7B). A combined logistic regression model achieved an AUC of 0.86 in the training set and 0.68 in the testing set (Figure 7C). In addition, linear regression with BMI as a continuous outcome showed that serum hexanoylglycine and LPC (16:0) levels were significantly associated with BMI (Figure S1).

### 3.8 Serum Levels of Hexanoylglycine and LPC (16:0) Are Associated With Blood Pressure

Finally, we assessed the relationships between serum hexanoylglycine and LPC (16:0) and blood pressure, a key clinical indicator of cardiometabolic risk. In correlation analyses, hexanoylglycine was inversely associated with SBP and DBP, whereas LPC (16:0) was positively associated with DBP (Figure 7D,E). We further conducted multivariable linear regression adjusting for age, sex, and BMI. Hexanoylglycine remained significantly associated with SBP but not DBP, while LPC (16:0) remained significantly associated with DBP (Table S3). Collectively, these findings suggest that hexanoylglycine and LPC (16:0) are associated with blood pressure profiles and may have biomarker potential for obesity‐related cardiometabolic dysfunction.

## 4 Discussion

Our integrated analysis identified hexanoylglycine and LPC (16:0) as candidate serum biomarkers associated with obesity and related cardiometabolic traits. Across observational analyses and MR, both biomarkers were linked to obesity‐related traits, immune‐related features, and blood pressure measures, supporting their potential value as biomarkers.

Hexanoylglycine was lower in obese participants, consistent with altered glycine related metabolism reported in obesity (Alves et al. 2019; Tan et al. 2024), and remained inversely associated with SBP after adjustment. In MR, genetically predicted hexanoylglycine showed a suggestive association with obesity risk and immune‐related traits, indicating possible immune involvement. The apparent discordance between lower measured levels in obesity and the direction of MR estimates may reflect differences between short‐term circulating concentrations and lifelong genetically proxied effects (Davies et al. 2018), obesity‐related metabolic perturbations affecting measured levels, and potential non‐linear or context‐dependent relationships. In addition, the discrepancy between the European‐based GWAS used for MR and our Chinese clinical cohort may partly reflect cross‐ancestry differences in genetic and environmental background. In particular, differences in genetic architecture across populations, including linkage disequilibrium patterns and allele frequencies, may affect the transferability of genetic instruments and contribute to heterogeneity in the direction and magnitude of effect estimates.

LPC (16:0) was higher in obese participants and remained positively associated with DBP after adjustment. Differences from prior reports (Bellot et al. 2023; Heimerl et al. 2014) may be driven by ancestry and phenotype differences, as well as lipidomics platform, processing, and annotation variability. Because LC–MS‐based lipidomics reports LPC (16:0) at the species level and may not resolve structural isomers, cross‐study comparisons should be made cautiously and replicated using harmonized phenotypes and targeted quantification. LPC species have been implicated in inflammatory signaling and immune cell responses (Fox et al. 2009; Liu et al. 2020), although the underlying mechanisms in obesity require further validation.

Several limitations should be noted. Our MR analyses primarily relied on European GWAS, which may limit transferability to our Chinese cohort; larger ancestry‐matched datasets and multi‐ethnic replication will be important. Differences in lipidomics platforms and annotation strategies may also contribute to heterogeneity across studies. The cohort was BMI‐defined, and the non‐obese group included both normal‐weight and overweight individuals, potentially increasing within‐group heterogeneity. Key clinical covariates were not comprehensively recorded, leaving the possibility of residual confounding, particularly for blood pressure‐ and lipid‐related associations. For example, BMI may not fully capture adiposity distribution or visceral fat burden, which are more closely related to metabolic dysfunction. In addition, incomplete information on lifestyle factors and medication use, including antihypertensive or lipid‐lowering treatment, may have influenced the observed associations. Given the modest sample size, ROC performance should be interpreted as exploratory and requires external validation. Finally, the proposed immune mediation pathways are hypothesis‐generating and require experimental confirmation.

Future work should prioritize replication in larger multi‐ethnic cohorts with detailed phenotyping and ancestry‐matched genetic instruments, prospective validation for incident obesity and cardiometabolic outcomes, and targeted LC–MS/MS assays enabling absolute quantification and standardized sample handling. Mechanistic studies are also needed to interrogate the immune‐related pathways suggested by the genetic and multi‐omics analyses.

## 5 Conclusions

In summary, we identified two serum biomarkers, hexanoylglycine and LPC (16:0), associated with obesity through integrated metabolomics, lipidomics, MR, and mediation analyses. Both biomarkers were significantly linked to obesity, with MR providing genetic evidence consistent with a potential causal role and suggesting immune cell involvement in their effects. These findings provide novel insights into potential pathways underlying obesity and highlight hexanoylglycine and LPC (16:0) as candidate biomarkers for clinical prediction and future mechanistic studies.

## Author Contributions

**Yongsheng Zhao:** data curation, writing – review and editing. **Yingting Wu:** data curation, formal analysis. **Wenli Zhao:** data curation, formal analysis. **Mengxi Jiang:** conceptualization, writing – review and editing, funding acquisition. **Mahan Si:** formal analysis, writing – original draft. **Xuan Liu:** data curation.

## Funding

This work was supported by the National Natural Science Foundation of China Grant (82270898) and the Youth Talent Training Program of Capital Medical University (B2404).

## Conflicts of Interest

The authors declare no conflicts of interest.

## Supporting information


**Figure S1:** Linear correlation analysis between BMI and differential biomarkers.

**Figure S2:** Instrument strength and variance explained for genetic instruments used in the two‐step mediation MR analysis.

**Figure S3:** Targeted quantification analysis of serum LPC (16:0) in non‐obese and obese individuals.

**Table S1:** GWAS ID Summary Table.

**Table S2:** Logistic result with age sensitivity.

**Table S3:** Multivariate linear regression of blood pressure adjusting for age, sex, and BMI.

**Table S4:** Optimized MRM parameters for the quantification of LPC (16:0) and its internal standard.

**Table S5:** Instrument strength metrics.

**Table S6:** Step‐specific MR estimates for each mediation path.

## Data Availability

De‐identified processed metabolomics and lipidomics matrices, differential feature lists and enrichment outputs, and summary results from MR and mediation analyses are available upon reasonable request (subject to ethics approval and a data‐use agreement if required). GWAS sources are listed in Supporting Information. Analysis scripts are available upon request.
