# Supplementary material for: Candidate Obesity Biomarkers Identified Through Multi‐Omics Analysis, Mendelian Randomization, and Mediation Analysis
Source: Food Sci Nutr. 2026 Apr 20;14(4):e71803. doi: 10.1002/fsn3.71803 (PMC13096563; doi:10.1002/fsn3.71803)
Supplement: Supplementary file 1 — Figure S1: Linear correlation analysis between BMI and differential biomarkers. [file FSN3-14-e71803-s003.docx]

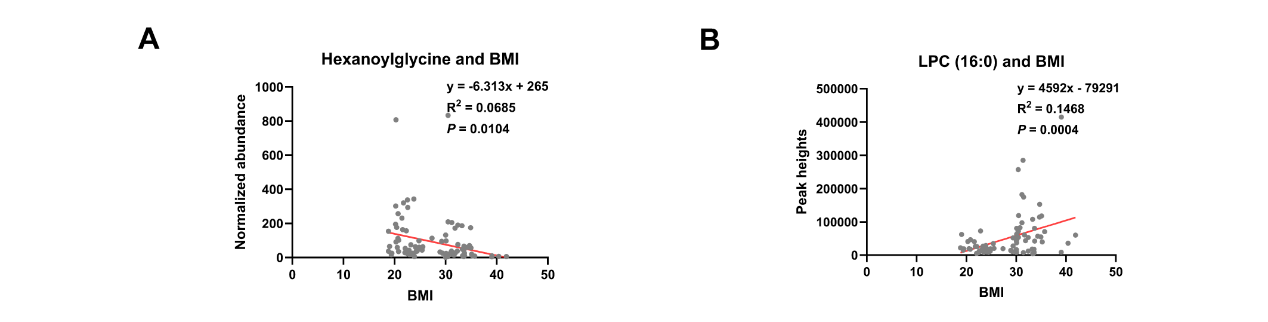
**Supplementary Figure 1. Linear correlation analysis between BMI and differential biomarkers.**

(**A, B**) Linear correlation analysis between continuous variable BMI and serum hexanoylglycine levels (left panel), and serum LPC(16:0) levels (right panel).
