# Supplementary material for: Candidate Obesity Biomarkers Identified Through Multi‐Omics Analysis, Mendelian Randomization, and Mediation Analysis
Source: Food Sci Nutr. 2026 Apr 20;14(4):e71803. doi: 10.1002/fsn3.71803 (PMC13096563; doi:10.1002/fsn3.71803)
Supplement: Supplementary file 2 — Figure S2: Instrument strength and variance explained for genetic instruments used in the two‐step mediation MR analysis. [file FSN3-14-e71803-s008.docx]

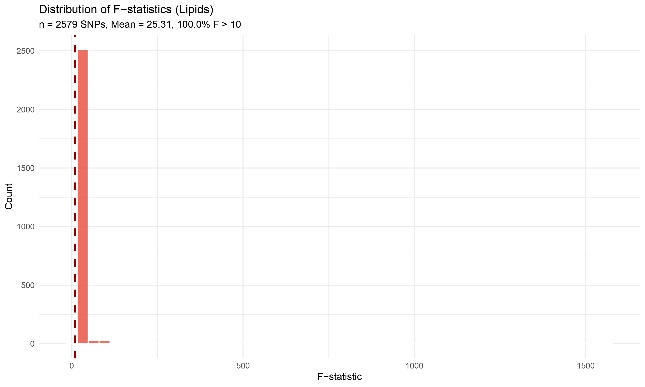
 **A B**


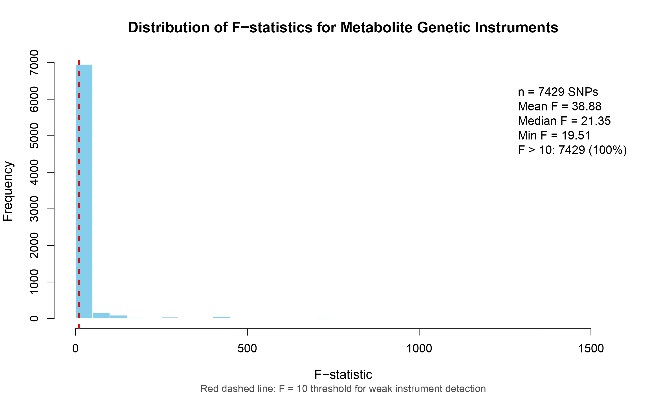


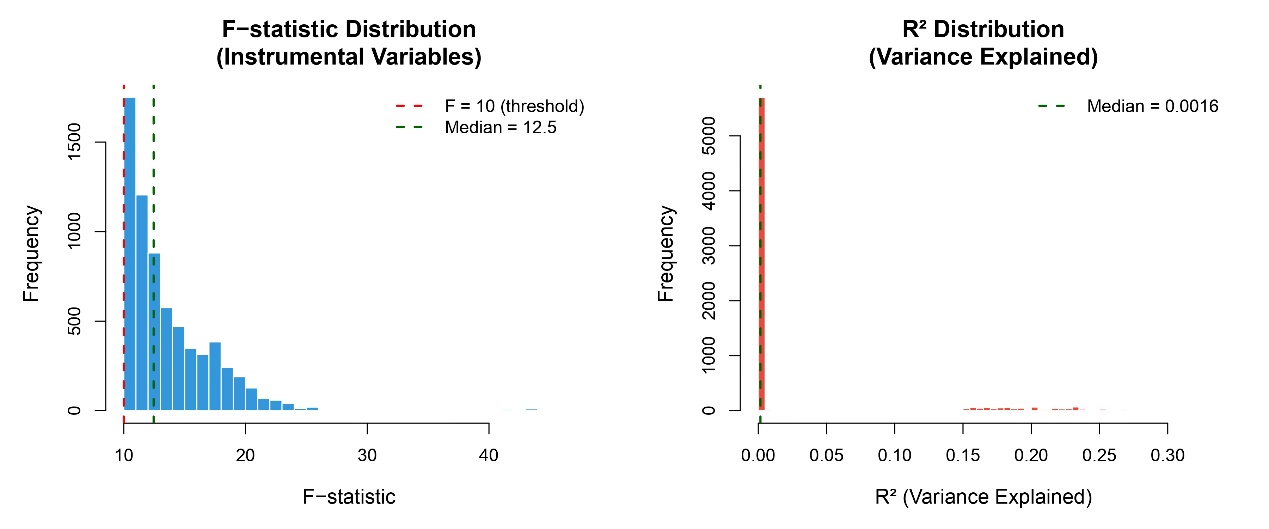
**C**

**Supplementary Figure 2. Instrument strength and variance explained for genetic instruments used in the two-step mediation MR analysis.**

**(A)** Distribution of SNP-level F-statistics for genetic instruments selected for metabolite exposures (p < 5×10⁻⁶).
**(B)** Distribution of SNP-level F-statistics for genetic instruments selected for lipid exposures (p < 5×10⁻⁶).
**(C)** Distributions of SNP-level F-statistics and variance explained (R²) across the instrument sets used in the mediation MR analyses. The dashed line indicates the conventional weak-instrument threshold (F = 10).
