# Supplementary material for: Candidate Obesity Biomarkers Identified Through Multi‐Omics Analysis, Mendelian Randomization, and Mediation Analysis
Source: Food Sci Nutr. 2026 Apr 20;14(4):e71803. doi: 10.1002/fsn3.71803 (PMC13096563; doi:10.1002/fsn3.71803)
Supplement: Supplementary file 3 — Figure S3: Targeted quantification analysis of serum LPC (16:0) in non‐obese and obese individuals. [file FSN3-14-e71803-s005.docx]

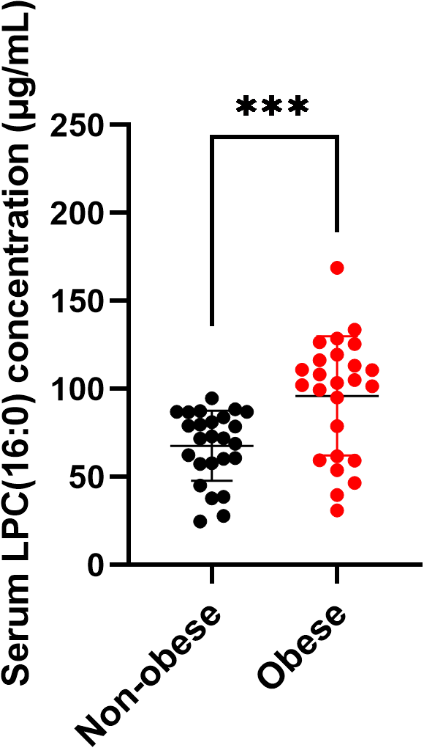


Supplementary Figure 3. Targeted LC-MS/MS quantification of serum LPC(16:0) in non-obese and obese individuals. Serum LPC(16:0) concentrations (μg/mL) in non- obese and obese individuals measured by targeted LC-MS/MS. Each dot represents one individual. n = 25 per group. Statistical significance was assessed using Student’s t-test.

***p < 0.001.
