# Supplementary material for: Candidate Obesity Biomarkers Identified Through Multi‐Omics Analysis, Mendelian Randomization, and Mediation Analysis
Source: Food Sci Nutr. 2026 Apr 20;14(4):e71803. doi: 10.1002/fsn3.71803 (PMC13096563; doi:10.1002/fsn3.71803)
Supplement: Supplementary file 7 — Table S4: Optimized MRM parameters for the quantification of LPC (16:0) and its internal standard. [file FSN3-14-e71803-s001.docx]

**Supplementary Table 4. Optimized MRM parameters for targeted LC-MS/MS quantification of LPC(16:0) and its deuterated internal standard.**

| **Compound** | **Precursor Ion (m/z)** | **Product Ion (m/z)** | **Collision Energy**  **(eV)** |
| --- | --- | --- | --- |
| LPC(16:0) | 496.2 | 184 | 28 |
| LPC-d31(16:0) | 527.3 | 184 | 26 |
